# Omicron BA.1 neutralizing antibody response following Delta breakthrough infection compared with booster vaccination of BNT162b2

**DOI:** 10.1186/s12879-023-08272-2

**Published:** 2023-05-04

**Authors:** Shohei Yamamoto, Kouki Matsuda, Kenji Maeda, Yusuke Oshiro, Natsumi Inamura, Tetsuya Mizoue, Maki Konishi, Junko S. Takeuchi, Kumi Horii, Mitsuru Ozeki, Haruhito Sugiyama, Hiroaki Mitsuya, Wataru Sugiura, Norio Ohmagari

**Affiliations:** 1 Department of Epidemiology and Prevention, Center for Clinical Sciences, National Center for Global Health and Medicine, 1-21-1, Toyama, Shinjuku-ku, Tokyo, 162-8655 Japan; 2 AIDS Clinical Center, National Center for Global Health and Medicine, Tokyo, Japan; 3 Japan Foundation for AIDS Prevention, Tokyo, Japan; 4 Division of Antiviral Therapy, Joint Research Center for Human Retrovirus Infection, Kagoshima University, Kagoshima, Japan; 5 Department of Refractory Viral Infection, Research Institute, National Center for Global Health and Medicine, Tokyo, Japan; 6 Department of Laboratory Testing, Center Hospital of the National Center for the Global Health and Medicine, Tokyo, Japan; 7 Department of Academic-Industrial Partnerships Promotion, Center for Clinical Sciences, National Center for Global Health and Medicine, Tokyo, Japan; 8 Infection Control Office, Center Hospital of the National Center for the Global Health and Medicine, Tokyo, Japan; 9 Center Hospital of the National Center for the Global Health and Medicine, Tokyo, Japan; 10 Center for Clinical Sciences, National Center for Global Health and Medicine, Tokyo, Japan; 11 Disease Control and Prevention Center, National Center for Global Health and Medicine, Tokyo, Japan

**Keywords:** SARS-CoV-2 breakthrough infection, Booster effect, Neutralizing antibody, Omicron variant, Delta variant

## Abstract

**Background:**

Longitudinal data are lacking to compare booster effects of Delta breakthrough infection versus third vaccine dose on neutralizing antibodies (NAb) against Omicron.

**Methods:**

Participants were the staff of a national research and medical institution in Tokyo who attended serological surveys on June 2021 (baseline) and December 2021 (follow-up); in between, the Delta-dominant epidemic occurred. Of 844 participants who were infection-naïve and had received two doses of BNT162b2 at baseline, we identified 11 breakthrough infections during follow-up. One control matched to each case was selected from boosted and unboosted individuals. We compared live-virus NAb against Wild-type, Delta, and Omicron BA.1 across groups.

**Results:**

Breakthrough infection cases showed marked increases in NAb titers against Wild-type (4.1-fold) and Delta (5.5-fold), and 64% had detectable NAb against Omicron BA.1 at follow-up, although the NAb against Omicron after breakthrough infection was 6.7- and 5.2-fold lower than Wild-type and Delta, respectively. The increase was apparent only in symptomatic cases and as high as in the third vaccine recipients.

**Conclusions:**

Symptomatic Delta breakthrough infection increased NAb against Wild-type, Delta, and Omicron BA.1, similar to the third vaccine. Given the much lower NAb against Omicron BA.1, infection prevention measures must be continued irrespective of vaccine and infection history while the immune evasive variants are circulating.

**Supplementary Information:**

The online version contains supplementary material available at 10.1186/s12879-023-08272-2.

## Key points

Symptomatic, not asymptomatic, SARS-CoV-2 breakthrough infection after the second BNT162b2 vaccination during the Delta-predominant wave enhanced neutralizing antibodies against Wild-type, Delta, and Omicron comparable to the three vaccine doses, although immunity against Omicron was much lower than Wild-type and Delta.

**Supplementary Information:**

The online version contains supplementary material available at 10.1186/s12879-023-08272-2.

## Background

Clinical trials showed that the mRNA-based vaccine was highly effective in reducing the risk of morbidity and mortality of coronavirus disease 2019 (COVID-19) [1]. The waning of vaccine-induced immunogenicity over time [2, 3] and the emergence of variants of concern (VOCs) with a high potential for immune evasion [4], however, have led to a marked increase in breakthrough infections and urged many countries to adopt the booster (third) vaccine campaign. As a result, the source of immunity against SARS-CoV-2 became diverse among people through their history of vaccination and infection. In this situation, there would be a need for quantitative data on immunogenicity according to infection/vaccination histories, which would help their decision on whether to receive a booster dose to prevent forthcoming variants.

A few studies showed that among infection-naïve two-dose vaccine recipients, neutralizing antibody (NAb) levels against VOCs, including the Omicron variant, were increased in persons who experienced Delta breakthrough infection or had received the third vaccine dose [5–7]. These studies, however, have some methodological limitations. No study considered potential modifiers of vaccine response such as age and sex in selecting controls or analyzing data and assessed the change of NAb between pre- and post-breakthrough infection. It thus remains elusive to what extent the NAb increased after breakthrough infection relative to the baseline. Additionally, few studies [6] analyzed the data by COVID-19 symptom, despite consistent evidence that higher antibody concentrations among patients with symptomatic infection than those with asymptomatic infection [8].

The objective of the present study was thus to assess live-virus NAb against the Wild-type, Delta, and Omicron variants among two-dose BNT162b2 vaccine recipients who experienced breakthrough infection during the Delta variant predominant wave, compared with unboosted and boosted infection-naïve individuals in a well-defined cohort of health care workers while addressing the issues mentioned above.

## Methods

### Study setting and case-control selection

We used data and samples from a repeated serological study among the staff of the National Center for Global Health and Medicine, Japan (NCGM). The detail of the study has been described elsewhere [9]. Written informed consent was obtained from all participants. The study was conducted according to the guidelines of the Declaration of Helsinki, and the procedure was approved by the NCGM Ethics Committee (approval number: NCGM-G-003598).

**Fig. 1:**
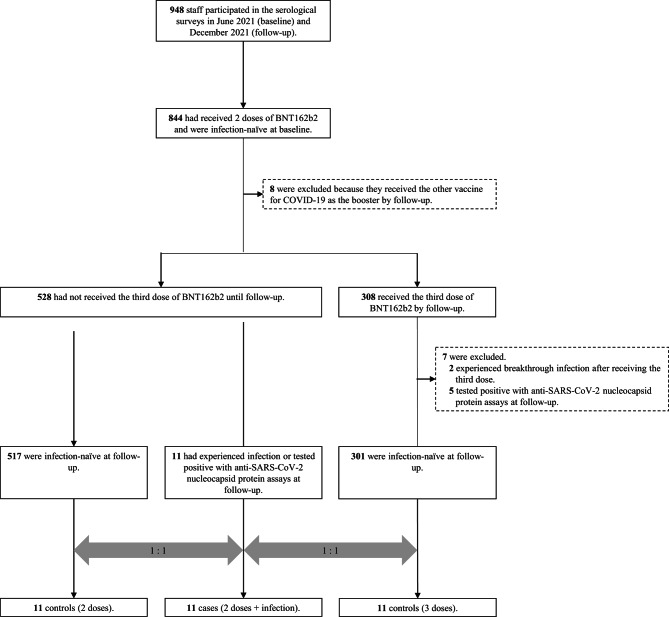
Flowchart for the case-control matching Infection-naïve were defined as having no history of COVID-19 and being negative with anti-SARS-CoV-2 nucleocapsid protein assays (both Abbott and Roche assays) Abbreviations: COVID-19, coronavirus disease 2019; SARS-CoV-2, severe acute respiratory syndrome coronavirus 2.

Of 948 participants who attended both baseline (June 2021) and follow-up (December 2021) surveys (Figs. 1), 844 had received two doses of BNT162b2 vaccine and were infection-naïve (i.e., no history of COVID-19 and negative with anti-SARS-CoV-2 nucleocapsid protein assays [both Abbott and Roche assays]) at baseline. Of these, we excluded 8 participants who received a vaccine other than BNT162b2 as the booster, leaving 836 included in the source population (528 had received two doses and 308 had received three doses by follow-up). Of those, we identified 11 patients who had the breakthrough infection during follow-up (7 cases were ascertained via in-house COVID-19 registry, and 4 cases were additionally identified with antibody test) and attended the follow-up survey without receiving the booster dose. All cases were assumed to be infected with the Delta variant, given its predominant circulation during the study period in Japan.

For each case, we selected infection-naïve controls from those who did not receive the third dose of the BNT162b2 and those who received the third dose during the study period, while matching demographic factors and baseline immunity status. In detail, we selected controls whose following characteristics were compatible with each case: sex, age (± 3 years), spike IgG antibody titer at baseline (± 10%), and the interval between the second vaccination and blood sampling at baseline. If multiple controls matched each case, one control was randomly selected from them.

### Neutralizing antibody testing

The NAb in serum was determined by quantifying the serum-mediated suppression of the cytopathic effect (CPE) of each SARS-CoV-2 strain in VeroE6_TMPRSS2_ cells [10, 11]. The obtained routes of the cells and each virus are described in Supplemental Text 1. Each serum sample was 4-fold serially diluted in a culture medium. The diluted sera were incubated with 100 50% tissue culture infectious dose (TCID_50_) of the virus at 37 °C for 20 min (final serum dilution range of 1:40 to 1:25000), after which the serum-virus mixtures were inoculated with VeroE6_TMPRSS2_ cells (1.0 × 10^4^/well) in 96-well plates. The SARS-CoV-2 strains used in these assays are as follows: a Wild-type, original strain (SARS-CoV-2^05−2 N^) [12], a Delta variant (SARS-CoV-2^TKYTK1734/2021^), and an Omicron BA.1 variant (SARS-CoV-2^TKYX00012/2021^). After culturing the cells for 3 to 5 days, the levels of CPE observed in SARS-CoV-2–exposed cells were determined using the WST-8 assay using the Cell Counting Kit-8 (Dojindo, Kumamoto, Japan). The serum dilution that gave 50% inhibition of CPE was defined as the 50% neutralization titer (NT_50_). Each serum sample was tested in duplicate, and the average value was used for analysis.

### SARS-CoV-2 antibody testing

We assessed anti–SARS-CoV-2 antibodies for all participants at baseline and follow-up and retrieved those data for the case-control subsets. We quantitatively measured antibodies against the receptor-binding domain (RBD) of the SARS-CoV-2 spike protein by using the AdviseDx SARS-CoV-2 IgG II assay (Abbott) (immunoglobulin [Ig] G [IgG]) and Elecsys^®^ Anti-SARS-CoV-2 S RUO (Roche) (including IgG). We also qualitatively measured antibodies against SARS-CoV-2 nucleocapsid protein using the SARS-CoV-2 IgG assay (Abbott) and Elecsys^®^ Anti-SARS-CoV-2 RUO (Roche), and used these data to exclude those with the possible infection before the baseline survey and to identify those who experienced breakthrough infection during the follow-up period. The sensitivity and specificity were 100% and 99.9%, respectively, for the Abbott assay [13], and 99.5% and 99.8%, respectively, for the Roche assay [14].

### Statistical analysis

We compared the characteristics between patients with breakthrough infection and their matched controls using the Kruskal-Wallis test or Fisher’s exact test. To examine the group difference of the changes in neutralizing and spike antibody levels from baseline to follow-up, we used a generalized estimating equation (GEE) with unstructured correlation structures and the robust variance estimator. Neutralizing and anti-spike antibody titers were log-transformed before analysis. Independent variables included were the matching indicator, time (baseline or follow-up), and interaction terms between group and time. The estimated effects of covariates were back-transformed and presented as ratios of geometric means. We repeated the GEE model to compare the neutralizing and spike antibody titers among the following three groups: those who had symptomatic breakthrough infections, asymptomatic breakthrough infections, or received three vaccinations. We also ran the GEE model with independent correlation structures to compare NAb titers against the Wild-type, Delta, and Omicron BA.1 within each of the following four groups: a group of all participants at baseline (n = 33) and three groups at follow-up (those who received only two doses (n = 11), those who experienced breakthrough infection (n = 11), and those who received three doses (n = 11)).

For analyses, values below or above the limit of detection (LOD) for NAb titers (NT_50_ < 40) and spike antibody titers with Roche assay (titer > 25,000 U/mL) were given the LOD value, respectively. Statistical analysis was performed using Stata version 17.0 (StataCorp LLC), and graphics were made by GraphPad Prism 9 (GraphPad, Inc). All P values were 2-sided, and P < 0.05 was considered statistically significant.

## Results

### Characteristics of breakthrough infection cases

Of the 11 patients of breakthrough infection, 55% were male, the median age was 26 (interquartile range [IQR]: 24–29) years, and the median body mass index was 21 (IQR: 20–22) kg/m^2^. Their major occupations were nurses (55%), allied healthcare professionals (18%), and doctors (5%), and 45% were engaged in COVID-19-related work. The median intervals from the second BNT162b2 vaccine to baseline and follow-up survey were 63 (IQR: 39–71) and 247 (IQR: 218–250) days, respectively. At baseline, the median spike antibody titers measured with Abbott and Roche assays were 3739 (IQR: 2435–9555) AU/mL and 935 (IQR: 762–1260) U/mL, respectively. The median NAb titers against Wild-type and Delta were 234 (IQR: 85–381) and 101 (IQR: 42–162) NT_50_, respectively, while no participant had detectable NAb against Omicron BA.1. These figures were similar between cases and matched control groups (Table 1) and between cases and the source population (Supplemental Table 1).

**Table 1:** Characteristics of the study participants

	SARS-CoV-2 infection and vaccination status at follow-up			
Characteristics	2 doses (N = 11)	2 doses + infection (N = 11)	3 doses (N = 11)	*P*
**Women**	6 (55)	6 (55)	6 (55)	1.00
**Age**, years	25 [24–30]	26 [24–29]	27 [25–29]	0.61
**Body mass index**, kg/m^2^	21 [18–21]	21 [20–22]	21 [19–24]	0.44
**Job**				
Doctor	3 (27)	1 ( 9)	2 (18)	0.65
Nurse	6 (55)	6 (55)	8 (73)	
Allied health professionals	2 (18)	2 (18)	1 ( 9)	
Others	0 ( 0)	2 (18)	0 ( 0)	
**COVID-19 related work**	6 (55)	5 (45)	4 (36)	0.90
**Intervals between vaccinations, infection, and surveys**				
Second dose to baseline, days	67 [40–74]	63 [39–71]	70 [67–73]	0.30
Second dose to follow-up, days	243 [221–251]	247 [218–250]	252 [249–256]	0.02
Baseline to follow-up, days	178 [177–181]	182 [179–184]	183 [181–187]	0.02
Third dose to follow-up, days	–	–	10 [8–10]	
Second dose to infection ^a^, days		124 [119–140]		
Baseline to infection ^a^, days		56 [53–65]		
Infection to follow-up ^a^, days	–	126 [113–130]	–	
**SARS-CoV-2 antibody titers at baseline**				
Spike IgG antibody (Abbott), AU/mL	3,671 [2,640 − 10,161]	3,739 [2,435-9,555]	3,811 [2,306-9,812]	0.99
Total antibody (Roche), AU/mL	1,282 [915-1,892]	935 [762-1,260]	1,258 [826-2,099]	0.34
NAb (Wuhan), NT_50_	290 [139–372]	234 [85–381]	182 [40–308]	0.44
NAb (Delta), NT_50_	168 [49–206]	101 [42–162]	76 [47–220]	0.74
NAb (Omicron BA.1), NT_50_	All < 40	All < 40	All < 40	–
**PCR-confirmed COVID-19 cases at follow-up**	0 (0)	0 (0)	7 (64)	–
**Seropositive against SARS-CoV-2 nucleocapsid protein at follow-up**	0 (0)	11 (100)	0 (0)	–
**Symptoms of breakthrough cases** ^b^				
Symptomatic	–	7 (64)	–	–
Asymptomatic	–	4 (36)	–	–
**Type of SARS-CoV-2 strain** ^c^				
Delta	–	4 (57)	–	–
Unknown (unmeasured)	–	3 (43)	–	–

Data are presented as median [interquartile range] for continuous measures and n (%) for categorical measures

^a^ The interval between breakthrough infection and follow-up survey was calculated for patients with PCR-confirmed COVID-19 (n = 7)

^b^ All patients with PCR-confirmed COVID-19 (n = 7) experienced any of the following symptoms: fever (n = 6), sore throat (n = 2), cough (n = 1), nasal discharge (n = 1), or malaise (n = 1). The remaining patients (n = 4), who were identified via an antibody test at follow-up, reported having no COVID-19 compatible symptoms during follow-up

^c^ The denominator is the number of patients with PCR-confirmed COVID-19 (n = 7)

*P* values for statistical significance were determined using the Kruskal-Wallis test or Fisher’s exact test

Abbreviations: AU, arbitrary units; COVID-19, coronavirus disease 2019; NT50, 50% neutralization titer; SARS-CoV-2, severe acute respiratory syndrome coronavirus 2

For 7 persons with a history of PCR-confirmed breakthrough infection, the median interval between the second dose and infection and between infection and follow-up survey was 124 (IQR: 119–140) and 126 (113–130) days, respectively. All these patients experienced any of the following symptoms: fever (n = 6), sore throat (n = 2), cough (n = 1), nasal discharge (n = 1), or malaise (n = 1), and were seropositive with anti-SARS-CoV-2 nucleocapsid protein assays at the follow-up survey. Data on virus strain were available for 4 of 7 (57%) cases (all were the Delta variant). The remaining 4 cases were identified via anti-SARS-CoV-2 N protein assays at the follow-up survey; all reported having no COVID-19 compatible symptoms during follow-up.

### Antibody titers waned over time and were boosted by breakthrough infection and the third dose

**Fig. 2:**
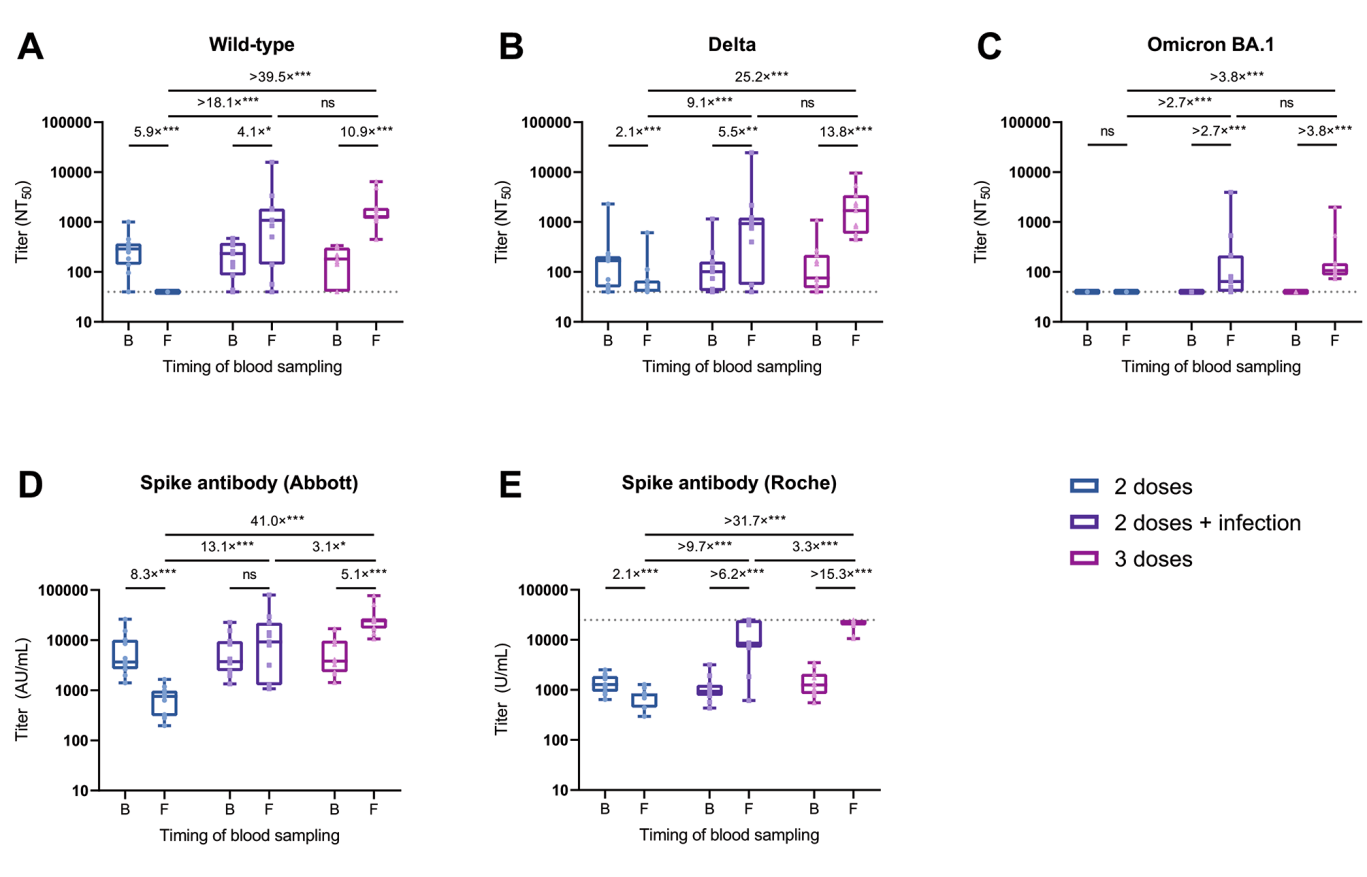
Change in the neutralizing and spike antibody titers in individuals who experienced breakthrough infection, received the booster vaccine, or were unboosted during follow-up Shown are NAb titers against the original Wild-type strain (**A**), the Delta variant (**B**), and the Omicron BA.1 variant (**C**) determined by 50% focus reduction neutralization test (FRNT_50_) using the serum at baseline and follow-up. Also shown are anti-spike antibody titers measured with the Abbott reagent (**D**) and the Roche reagent (**E**) at baseline and follow-up. Box plots show the median, interquartile range, and full range. The dushed horizontal lines indicate the LOD in the present analysis (NT_50_ < 40 in FRNT_50_ and U/mL > 25,000 in Roche assay) The fold-change values are estimated ratios of geometric means for antibody titers based on the GEE model (ns: not significant; *P < 0.05; **P < 0.01; ***P < 0.001) Abbreviations: AU, arbitrary units; B, baseline; F, follow-up; GEE, generalized estimating equation; LOD, limits of detection; NT_50_, 50% neutralization titer; SARS-CoV-2, severe acute respiratory syndrome coronavirus 2.

During the follow-up, NAb titers against Wild-type showed a 4.1-fold and 10.9-fold increase in those who experienced breakthrough infection and those who received the third dose, respectively. In contrast, it became undetectable in all unboosted infection-naïve individuals (Fig. 2A). At the follow-up survey, persons with breakthrough infections had a 2.2-fold lower, albeit statistically not significant, geometric mean titers (GMT) against Wild-type (723, 95% CI: 261–2001) than the third-dose vaccine recipients (1580, 95% CI: 1043–2395). The NAb titer against Delta was 2.1-fold decreased in those who received only two doses, whereas it showed 5.5-fold and 13.8-fold increases in those with breakthrough infection and those with three doses, respectively (Fig. 2B). Consequently, persons with breakthrough infection had a 2.8-fold lower, albeit statistically not significant, GMT against Delta (GMT: 564, 95% CI: 187–1703) than those who received the third dose (GMT: 1563, 95% CI: 873–2801) at the follow-up survey. The NAb titers against Omicron BA.1, which was not detectable in all groups at baseline, was quantifiable in two-thirds of those who experienced breakthrough infection (GMT: 108, 95% CI: 72–163) and all persons who received the third dose (GMT: 152, 95% CI: 101–228) (Fig. 2C).

Mean spike antibody titer with Abbott assay showed an 8.3-fold decrease in those who did not receive the booster dose, no change in those with breakthrough infections, and a 5.1-fold increase in the third vaccine recipients (Fig. 2D). As a consequence, persons with breakthrough infection had a 3.1-fold lower mean titer than those who received the third dose at follow-up. Mean spike antibody titer with Roche assay showed a 2.1-fold decrease in those who did not receive the booster dose, whereas it showed at least 6.2-fold and 15.3-fold increase in those who experienced breakthrough infection and the recipients of the third dose, respectively (Fig. 2E). At follow-up, persons with breakthrough infection had a 3.3-fold lower mean titer than the third-dose vaccine recipients.

### Symptomatic, but not asymptomatic, breakthrough infection cases had NAb titers comparable to the third dose recipients

**Fig. 3:**
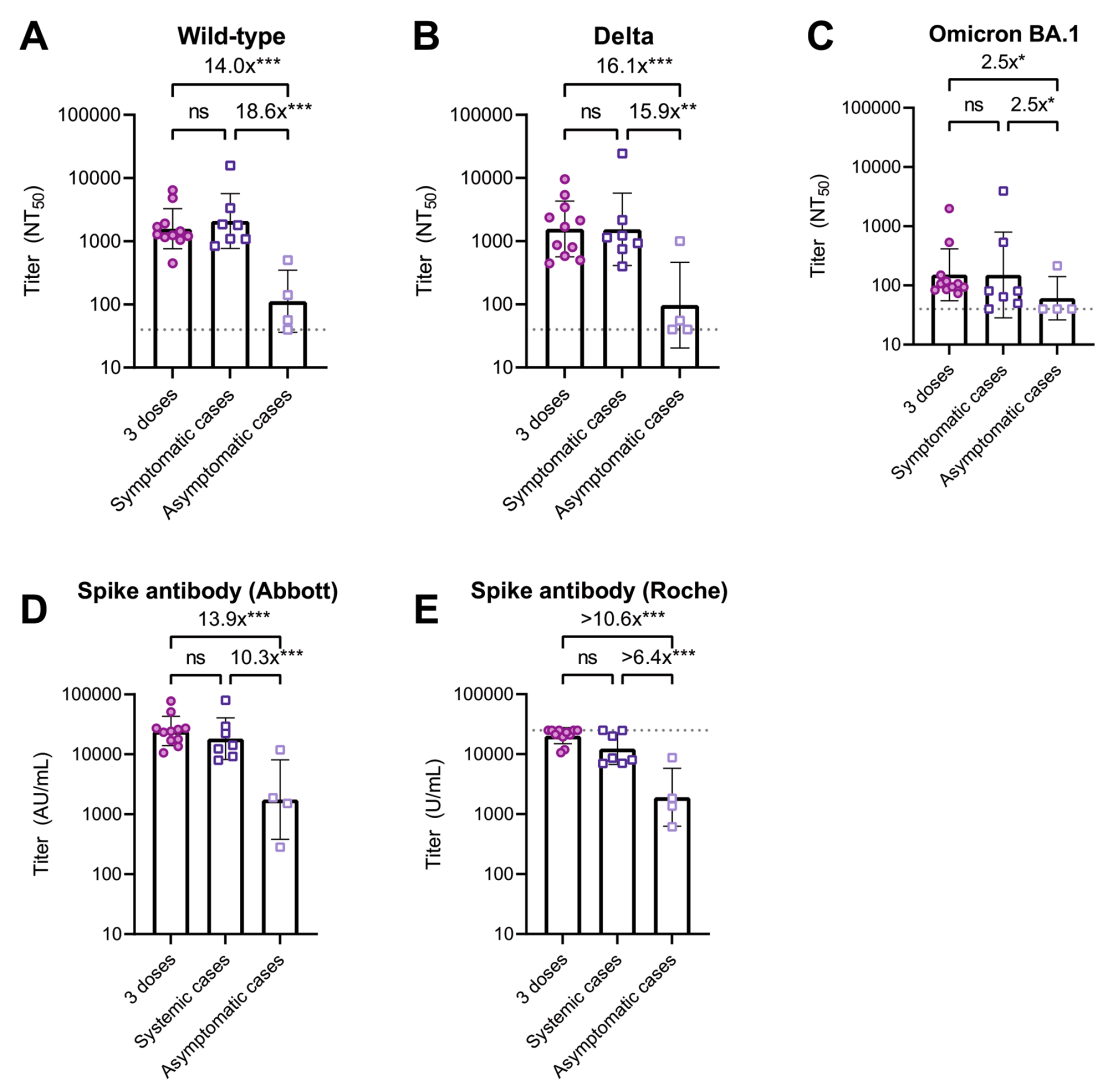
Neutralizing and spike antibody titers after three vaccine doses, symptomatic and asymptomatic breakthrough infections Shown are NAb titers against the original Wild-type strain (**A**), the Delta variant (**B**), and the Omicron BA.1 variant (**C**) determined by 50% focus reduction neutralization test (FRNT_50_) using the serum at follow-up. Also shown are anti-spike antibody titers measured with the Abbott reagent (**D**) and the Roche reagent (**E**) using the serum at follow-up Patients with PCR-confirmed infection were all symptomatic (n = 7), while those with seropositive on any of anti-SARS-CoV-2 nucleocapsid protein assays (Abbott or Roche assays) at follow-up were all asymptomatic (n = 4) The bars indicate geometric mean titers, and I-shaped bars indicate its geometric standard deviations. The dushed horizontal lines indicate the LOD for FRNT_50_ (NT_50_ < 40) and Roche assay (U/mL > 25,000) in the present analysis Statistical significance was determined by Kruskal-Wallis and Dunn’s multiple comparison test (ns: not significant; *P < 0.05; **P < 0.01; ***P < 0.001) Abbreviations: AU, arbitrary units; COVID-19, coronavirus disease 2019; LOD, limits of detection; NT50, 50% neutralization titer; SARS-CoV-2, severe acute respiratory syndrome coronavirus 2.

All types of NAb titer were substantially increased in persons with symptomatic breakthrough infection (3.8 to 17.7 folds), whereas the titer against Wild-type showed a 3.1-fold decrease and those against Delta and Omicron BA.1 did not increase in those with asymptomatic breakthrough infection (Figure S1). At follow-up, persons with a history of symptomatic infection had GMT against Wild-type of 2093, Delta of 1542, and Omicron BA.1 of 151, comparable to those of the third vaccine recipients (1580, 1563, and 152 in sequential order; Fig. 3).

### Neutralizing capacities against Omicron BA.1 was markedly lower than those against Wild-type and Delta after breakthrough infection or third dose of vaccine

**Fig. 4:**
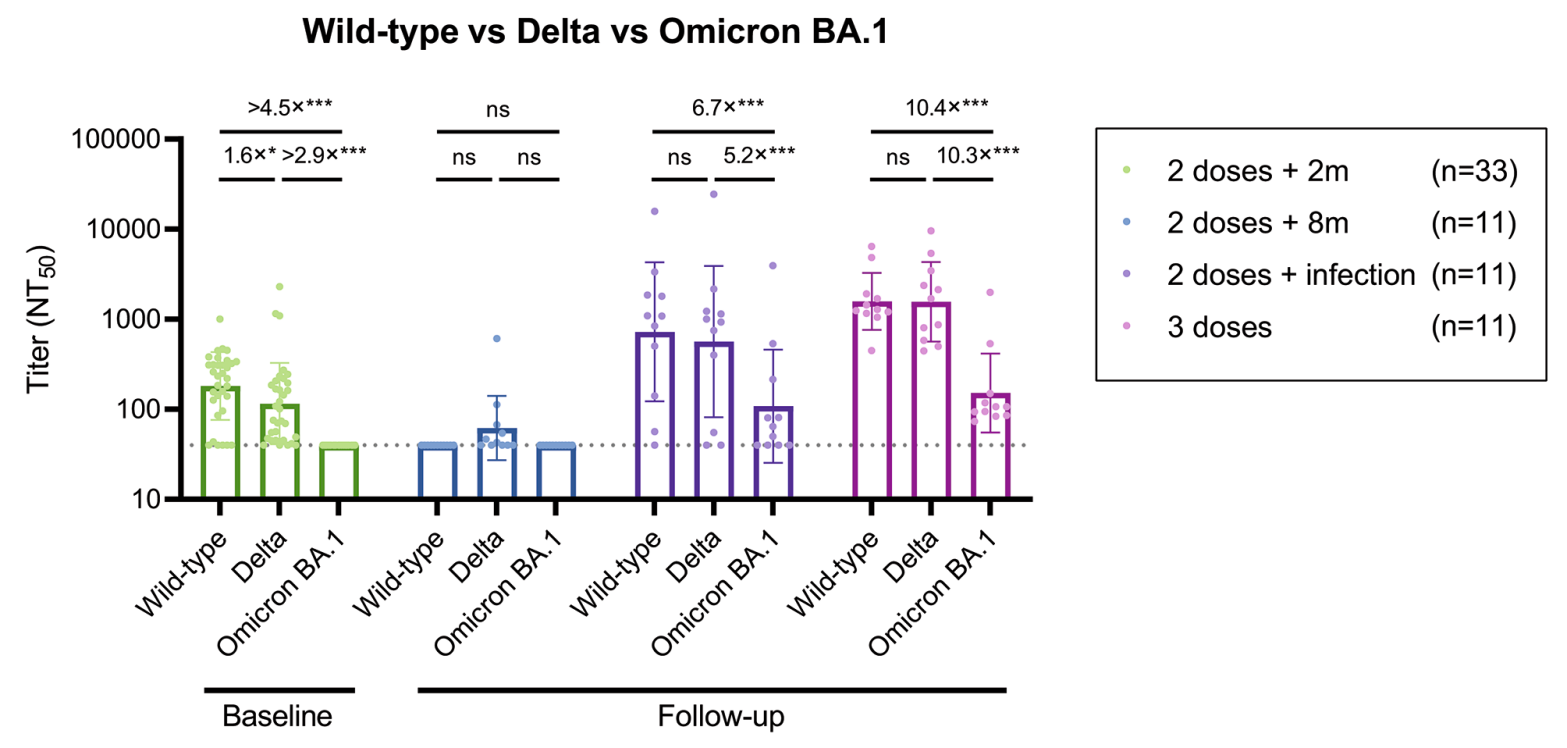
Comparison of neutralizing antibody titers against Wild-type, Delta, and Omicron BA.1 strains Shown are the NAb titers against the original Wild-type strain, the Delta variant, and the Omicron BA.1 variant as determined by the 50% focus reduction neutralization test (FRNT_50_). The bars indicate geometric mean titers, and I-shaped bars indicate its geometric standard deviations. The dushed horizontal lines indicate the LOD (NT_50_ titer < 40) Statistical significance was determined by the GEE model (ns: not significant; *P < 0.05; **P < 0.01; ***P < 0.001) Abbreviations: GEE, generalized estimating equation; LOD, limits of detection; NT_50_, m: months, 50% neutralization titer; 2 doses + 2 m, two months following the two doses; 2 doses + 8 m, eight months following the two doses; 95% CI, 95% confidence interval.

At baseline (all serum samples combined), most had a quantifiable NAb against Wild-type (85%) and Delta (89%); the geometric mean titer against Delta was 1.6-fold lower than that against Wild-type. None had a quantifiable NAb titer against Omicron BA.1 (Fig. 4). At follow-up, NAb titer against Omicron BA.1 was markedly lower than that against Wild-type and Delta in persons who experienced the breakthrough infection (6.7- and 5.2-fold, respectively) and those who completed the third dose (10.4- and 10.3-fold, respectively) (Fig. 4).

## Discussion

In this matched longitudinal study among infection-naïve individuals who received two doses of BNT162b2, the live-virus NAb titers against Wild-type, Delta, and Omicron BA.1 were increased among persons who were infected during the Delta-dominant epidemic period. The increase of NAb was prominent in persons with symptomatic, but not asymptomatic, breakthrough infection and was comparable to that in the third-dose vaccine recipients.

Our finding of an increase in cross-reactive NAb against Wild-type and Omicron after breakthrough infection during the Delta wave is consistent with previous reports [5–7]. With the rigorous matching of background factors, the use of paired serum samples, and the assessment of NAb against live viruses, the present study adds to confirmatory evidence in the literature regarding the role of Delta breakthrough infection in immunological cross-reactivity to Omicron. Nevertheless, we should note that their NAb titers against Omicron were much lower than those against Wild-type and Delta, probably due to frequent spike mutations in the Omicron variant [15]. This result is compatible with observational data showing frequent reinfections with Omicron among those with a history of infection with former SARS-CoV-2 strains, including Delta [16], and lowered effectiveness of three BNT162b2 doses against Omicron infection [17, 18].

The severity of COVID-19 has been suggested to correlate with post-infection immunogenicity [8]. In our analysis, NAb titers against Wild-type and Delta were increased in persons with COVID-19 compatible symptoms at breakthrough infection but not in those with asymptomatic infection (detected with antibody test only). This result is in line with a study among patients who recovered from Delta infection [6], reporting higher NAb titers against Wild-type in those with moderate to severe symptoms than in those with no or mild symptoms. More importantly, we found that among patients who were infected during the high circulation of Delta, the proportion of having a detectable NAb titer against Omicron was much higher among persons who had any symptom than those who had no symptom (86% vs. 25%), suggesting that cross-reactivity induced by Delta infection against the Omicron variant also depends on the symptom.

The limitations of this study should be acknowledged. First, the interval from SARS-CoV-2 exposure (vaccination or infection) to blood sampling markedly differed between the third vaccine recipients (median 10 [IQR: 8–11] days) and the patients with PCR-confirmed breakthrough infection (median 126 [IQR: 113–130] days). Accordingly, antibody titers might be still on the rise in the former, while they may reflect waning over time in the latter. Second, viral sequence data were available for only 4 (57%) patients among PCR-confirmed COVID-19 cases. Nevertheless, we can reasonably assume that the remaining breakthrough infections are also due to the Delta variant, which accounted for more than 90% of sequenced COVID-19 samples in Japan during the follow-up (June to December 2021) [19]. Third, we did not assess cellular immune response, another important mechanism for preventing severe COVID-19 [20]. Finally, study participants were predominantly lean Japanese (median [IQR] body mass index of 21 [19–22] kg/m^2^). Caution should be exercised in generalizing the present findings to the populations with different backgrounds.

## Conclusions

In summary, recipients of two doses of BNT162b2 who subsequently experienced symptomatic breakthrough infection (possibly with Delta) showed a substantial increase in NAb titers against Wild-type, Delta, and, to a lesser extent, Omicron BA.1, similar to those who received the third vaccine dose. The results may help those who experienced breakthrough infection in their decision-making whether to receive the booster vaccine. Given their markedly lower cross-reactive NAb titers against Omicron than other virus types, however, infection prevention measures must be continued, irrespective of SARS-CoV-2 infection or vaccination history.

## Electronic supplementary material

Below is the link to the electronic supplementary material.

Supplementary Material 1

## Data Availability

The datasets generated during and/or analysed during the current study are available from the corresponding author on reasonable request.

## List of Abbreviations

CPE: Cytopathic effect
COVID-19: Coronavirus disease 2019
GEE: Generalized estimating equation
IQR: Interquartile range
IgG: Immunoglobulin G
LOD: Limit of detection
NCGM: National Center for Global Health and Medicine, Japan
NAb: Neutralizing antibodies
RBD: Receptor-binding domain
SARS-CoV-2: Severe acute respiratory syndrome coronavirus 2
VOCs: Variants of concern
NT50: 50% neutralization titer
TCID50: 50% tissue culture infectious dose
