# Supplementary material for: Omicron BA.1 neutralizing antibody response following Delta breakthrough infection compared with booster vaccination of BNT162b2
Source: BMC Infect Dis. 2023 May 4;23:282. doi: 10.1186/s12879-023-08272-2 (PMC10157119; doi:10.1186/s12879-023-08272-2)
Supplement: Supplementary file 1 — Supplementary Material 1 [file 12879_2023_8272_MOESM1_ESM.docx]

**Supplementary Appendix**

Contents

[**Supplemental Text 1.** The sources of cells and virus strains 2](#_Toc132308397)

[**Supplemental Table 1.** Characteristics of the breakthrough infection cases and non-infected controls among the source cohort 3](#_Toc132308398)

[**Supplemental Figure 1**. Change in the neutralizing and spike antibody titers against SARS-CoV-2 before and after breakthrough infections stratified by the presence of symptoms 4](#_Toc132308399)

# **Supplemental Text 1.** The sources of cells and virus strains

VeroE6_TMPRSS2_ cells were obtained from the Japanese Collection of Research Bioresources (JCRB) Cell Bank (Osaka, Japan) and maintained in Dulbecco’s Modified Eagle Medium (DMEM) supplemented with 10% FCS, 100 µg/ml of penicillin, 100 µg/ml of streptomycin, and 1 mg/mL of G418. As the Wild-type strain, the SARS-CoV-2 NCGM-05-2N strain (SARS-CoV-2^05-2N^) was isolated from nasopharyngeal swabs of a patient with COVID-19 who was admitted to the NCGM hospital in the early phase of the COVID-19 pandemic in 2020. Two clinically isolated SARS-CoV-2 mutant strains, which were provided by the Tokyo Metropolitan Institute of Public Health, Tokyo, Japan, were used in the current study: the B.1.617.2 (Delta) strain [hCoV-19/Japan/TKYK01734/2021 (SARS-CoV-2^1734^, GISAID Accession ID; EPI_ISL_2080609)], the B.1.1.529; BA.1 (Omicron BA.1) strain [hCoV-19/Japan/TKYX00012/2021 (SARS-CoV-2^TKYX00012/2021^, GISAID Accession ID; EPI_ISL_8559478)]. Each variant was confirmed to contain each VOC-specific amino acid substitution before the assays conducted in the present study.

# **Supplemental Table 1.** Characteristics of the breakthrough infection cases and non-infected controls among the source cohort

| **Characteristics** | **Breakthrough infection cases**  **(N=11)** | **Non-infected controls**  **(N=825)** | ***P*** |
| --- | --- | --- | --- |
| **Women** | 6 (55) | 626 (76) | 0.15 |
| **Age**, years | 27 [25-29] | 29 [25-37] | 0.46 |
| **Body mass index**, kg/m^2^ | 21 [20-22] | 21 [19-22] | 0.51 |
| **Job** |  |  |  |
| Doctor | 1 ( 9) | 114 (14) | 0.15 |
| Nurse | 6 (55) | 540 (66) |  |
| Allied health professionals | 2 (18) | 138 (17) |  |
| Others | 2 (18) | 33 ( 4) |  |
| **COVID-19 related work** | 5 (45) | 337 (41) | 1.00 |

Data are presented as median [interquartile range] for continuous measures and n (%) for categorical measures.

*P* values for statistical significance were determined using the Kruskal-Wallis test or Fisher’s exact test.

Abbreviations: COVID-19, coronavirus disease 2019.

# **Supplemental Figure 1**. Change in the neutralizing and spike antibody titers against SARS-CoV-2 before and after breakthrough infections stratified by the presence of symptoms

Panel **A** shows neutralizing antibody titers against the original Wild-type strain, the Delta variant, and the Omicron BA.1 variant determined by 50% focus reduction neutralization test (FRNT_50_) using the serum at baseline and follow-up. Panel **B** shows anti-spike antibody titers measured with the Abbott reagent (AU/mL) and the Roche reagent (U/mL) at baseline and follow-up.

Symptomatic cases correspond to those with positive on PCR testing (n=7), while asymptomatic cases correspond to those with seropositive on any of the anti-SARS-CoV-2 nucleocapsid protein assays (Abbott or Roche assays) at the follow-up survey (n=4).

Box plots show the median, interquartile range, and full range. The dushed horizontal lines indicate the LOD in the present analysis (NT_50_<40 in FRNT_50_).

The fold-change values are estimated ratios of geometric means for antibody titers based on the GEE model (ns: not significant; *P<0.05; **P<0.01; ***P<0.001).

Abbreviations: AU, arbitrary units; B, baseline; F, follow-up; GEE, generalized estimating equation; LOD, limits of detection; NT_50_, 50% neutralization titer; SARS-CoV-2, severe acute respiratory syndrome coronavirus 2.
